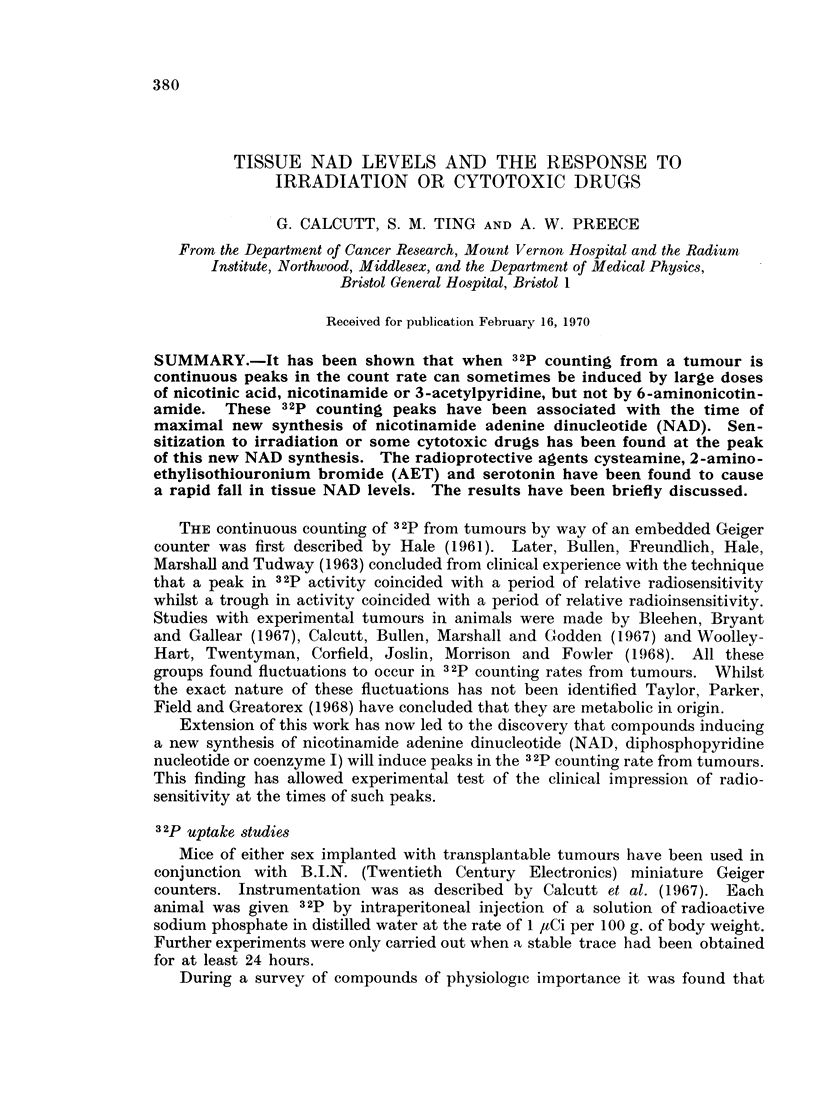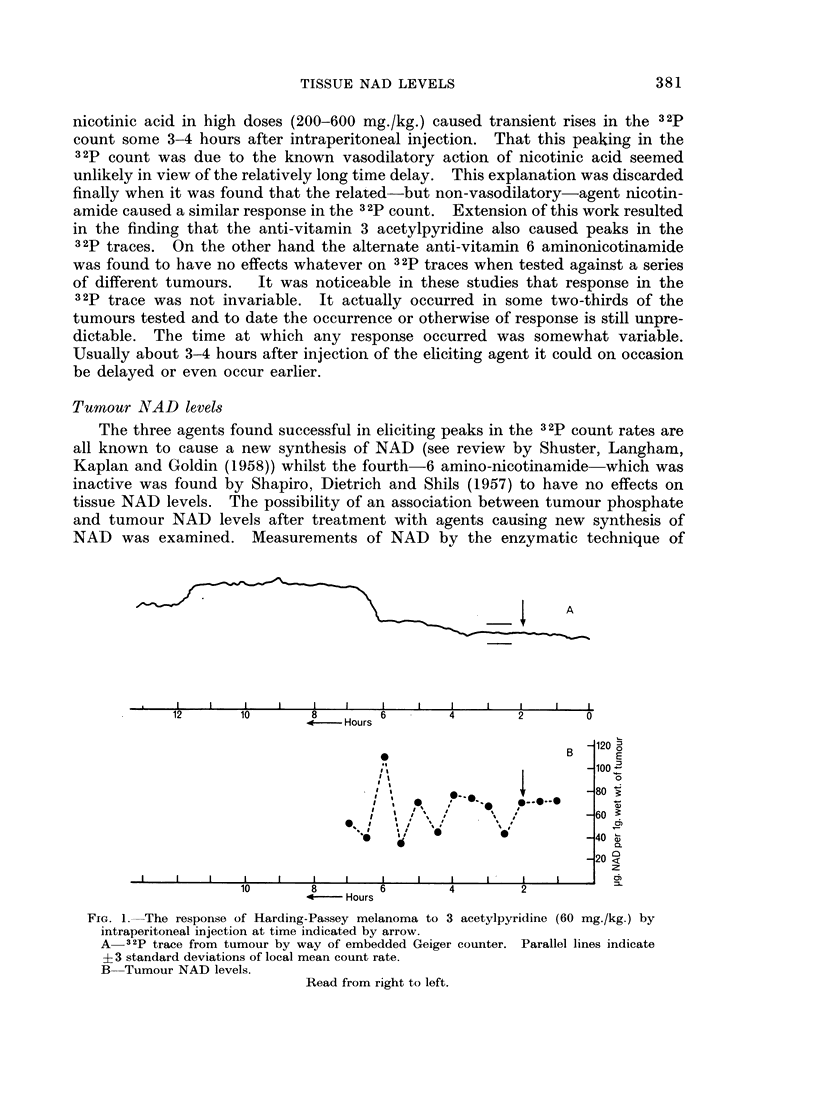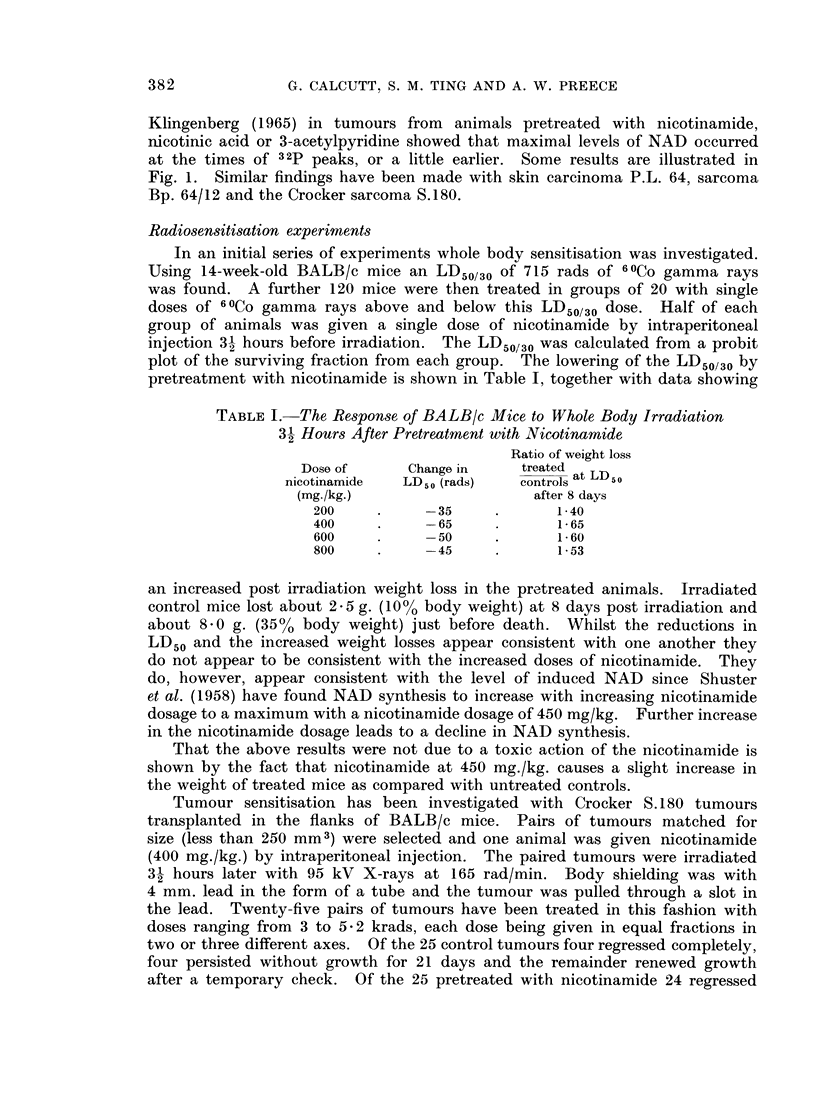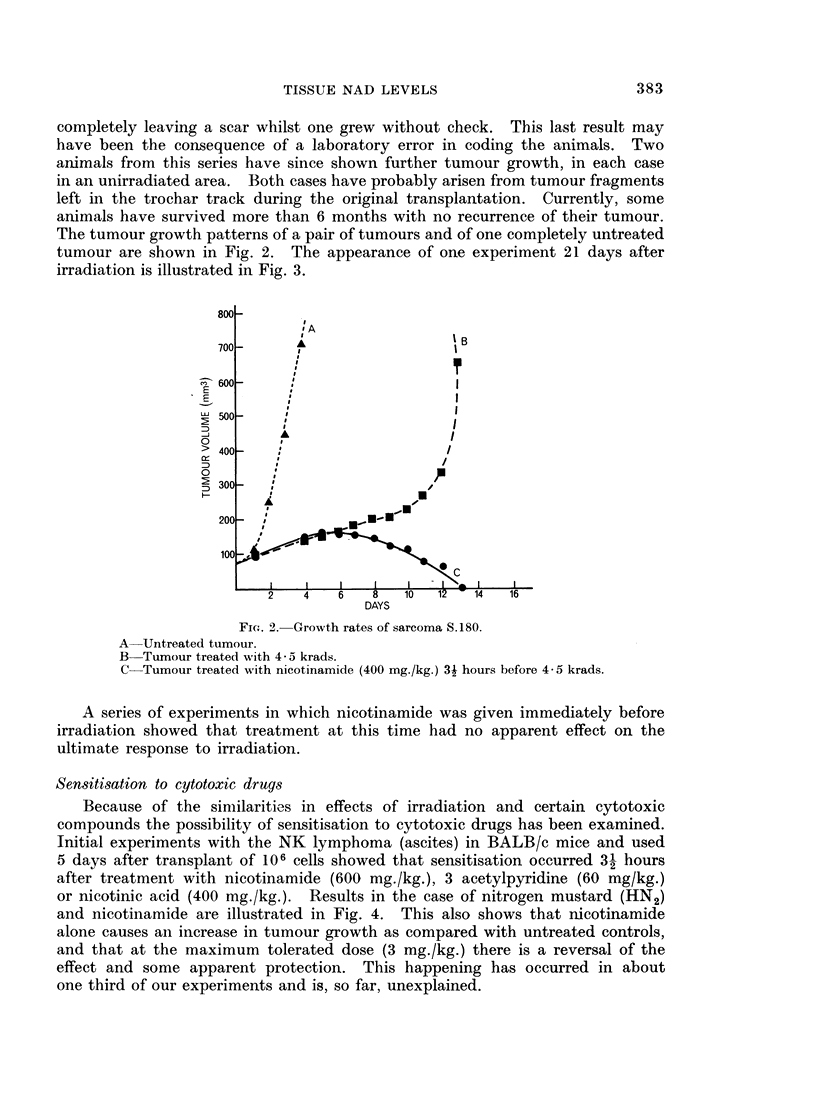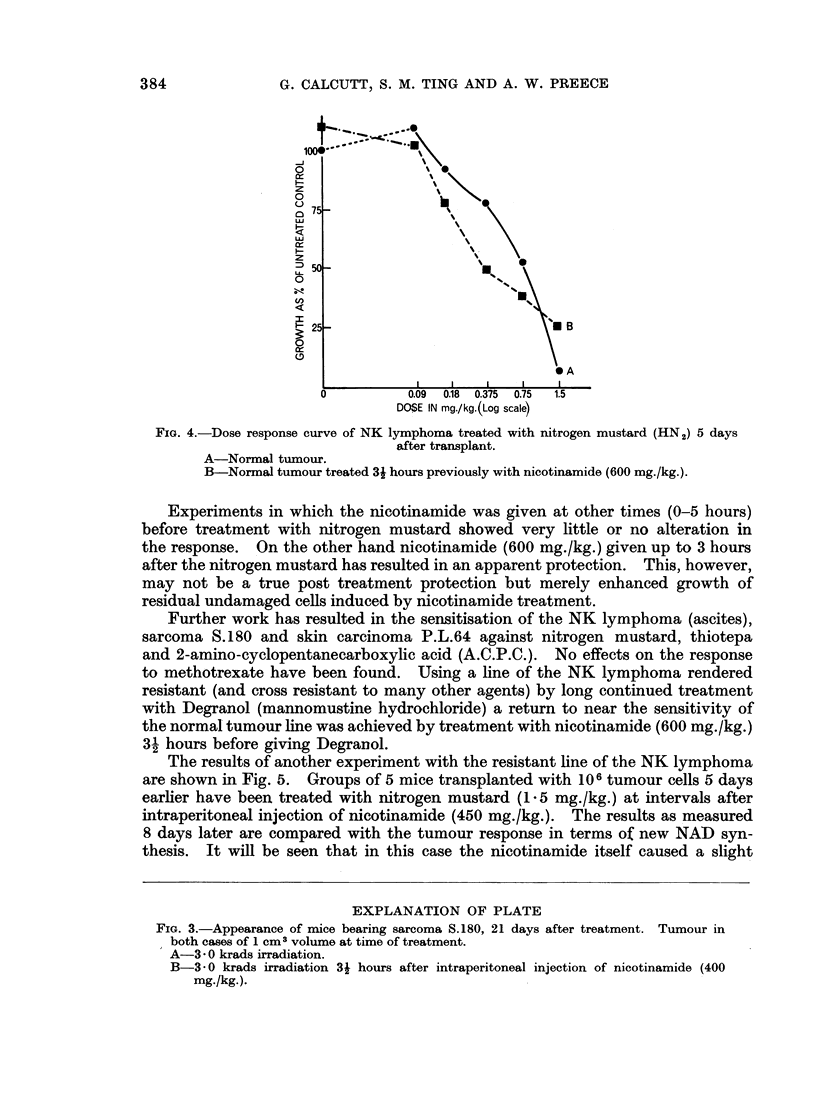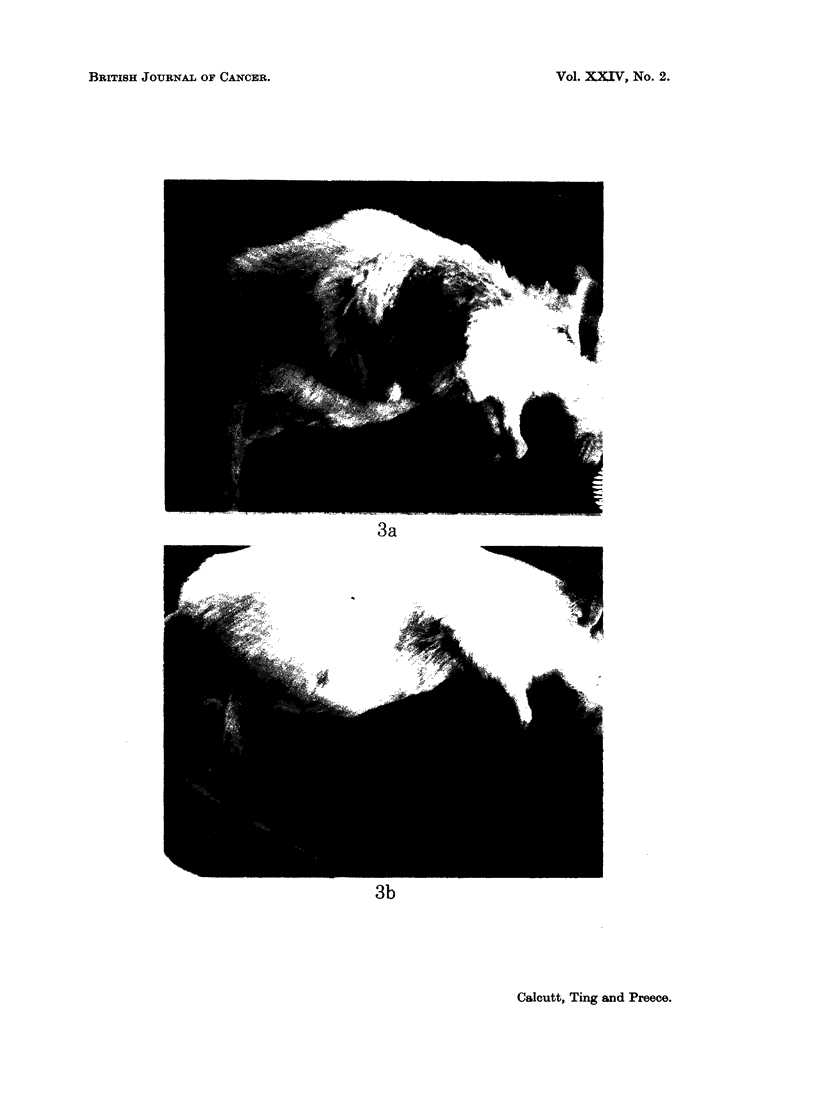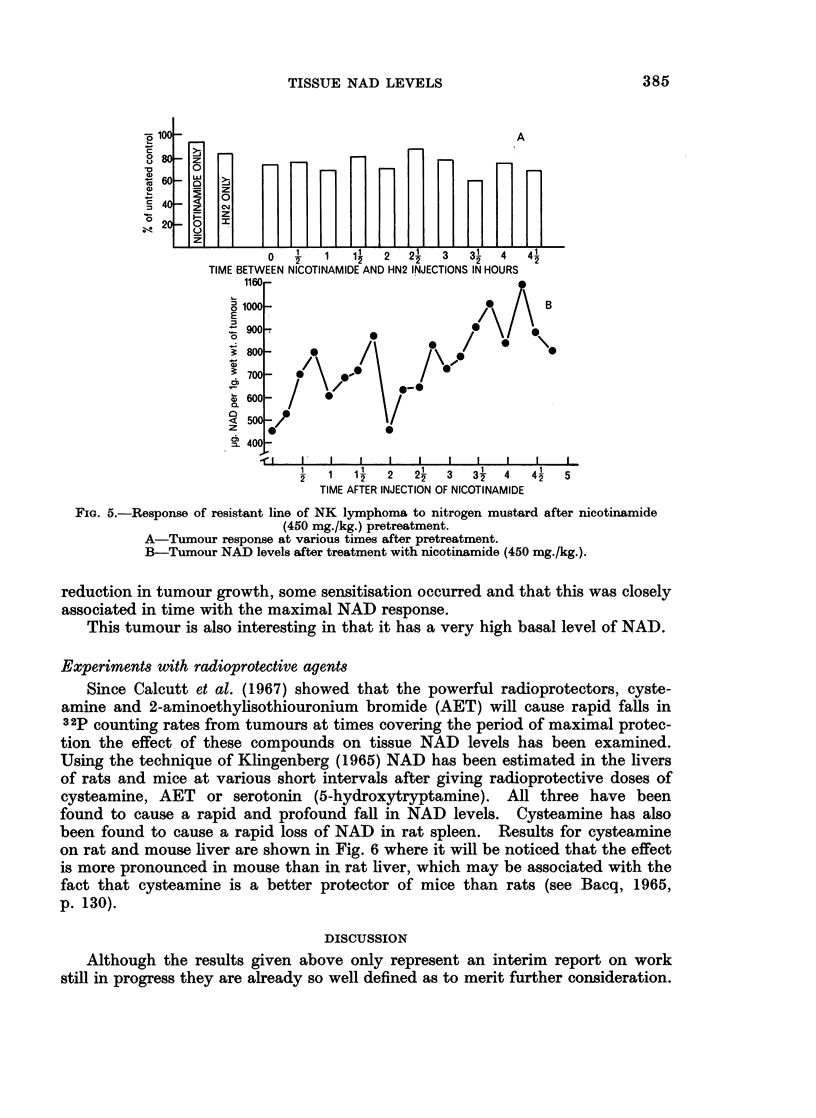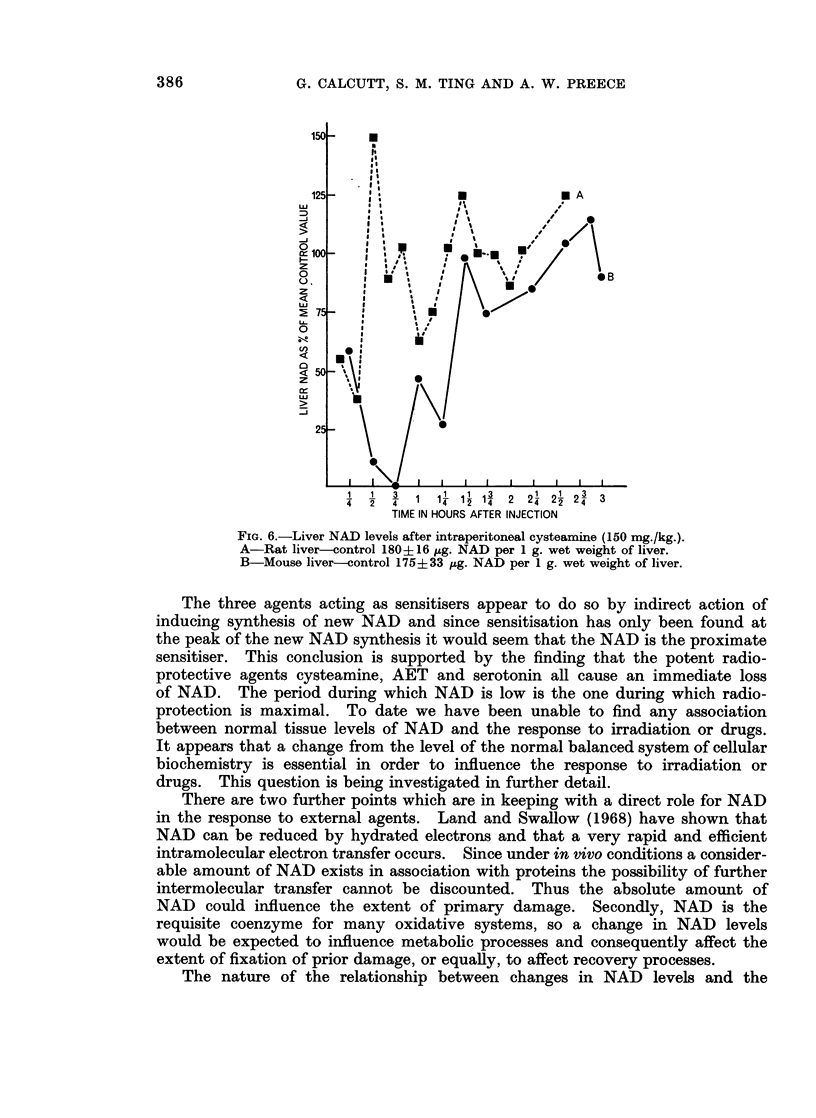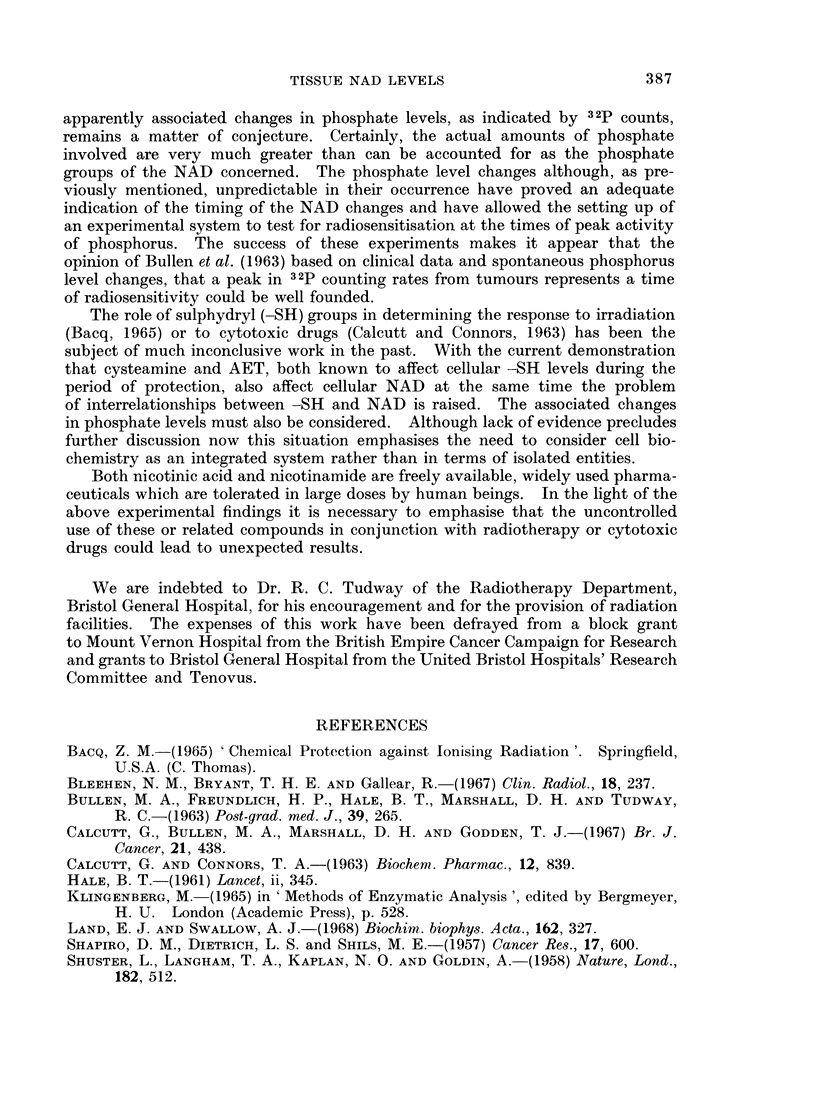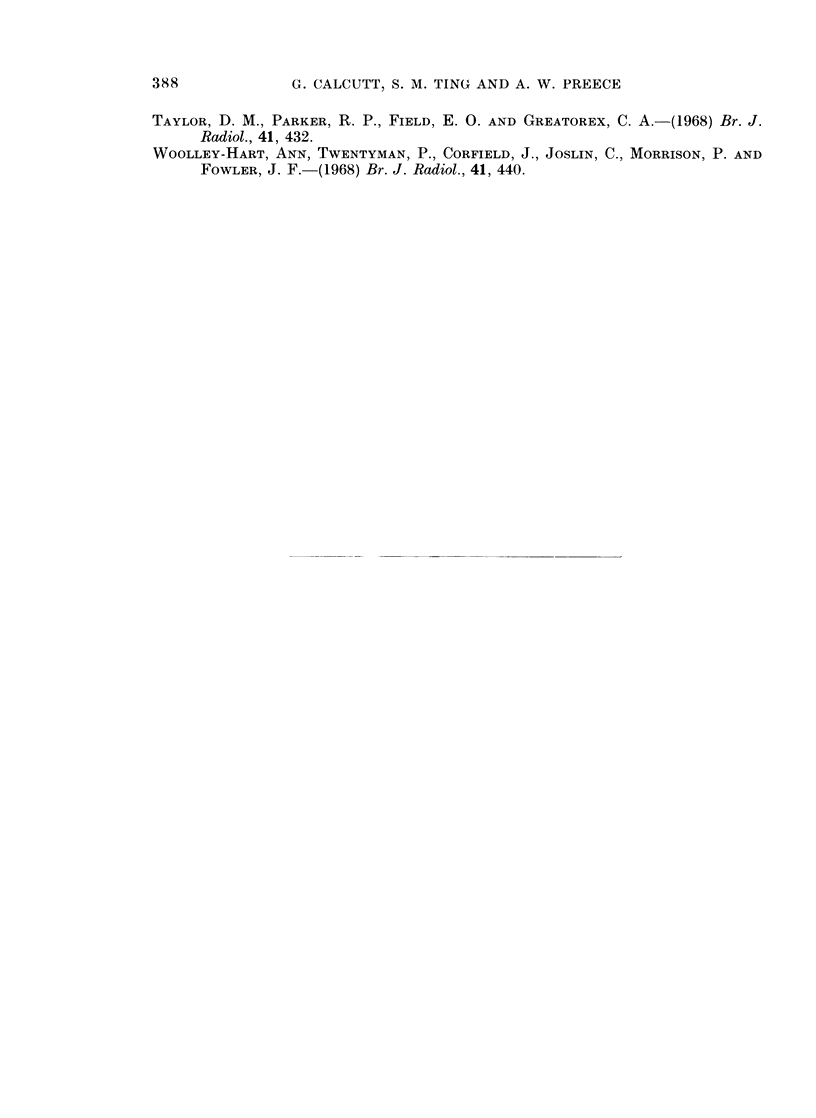# Tissue NAD Levels and the Response to Irradiation or Cytotoxic Drugs

**DOI:** 10.1038/bjc.1970.44

**Published:** 1970-06

**Authors:** G. Calcutt, S. M. Ting, A. W. Preece

## Abstract

**Images:**


### TISSUE NAD LEVELS AND THE RESPONSE TO IRRADIATION OR CYTOTOXIC DRUGS

G. CALCUTT, S. M. TING AND A. W. PREECE

*From the Department of Cancer Research, Mount Vernon Hospital and the Radium Institute, Northwood, Middlesex, and the Department of Medical Physics, Bristol General Hospital, Bristol 1*

Received for publication February 16, 1970

**SUMMARY.—It has been shown that when $^{32}P$ counting from a tumour is continuous peaks in the count rate can sometimes be induced by large doses of nicotinic acid, nicotinamide or 3-acetylpyridine, but not by 6-aminonicotinamide. These $^{32}P$ counting peaks have been associated with the time of maximal new synthesis of nicotinamide adenine dinucleotide (NAD). Sensitization to irradiation or some cytotoxic drugs has been found at the peak of this new NAD synthesis. The radioprotective agents cysteamine, 2-aminoethylisothiouronium bromide (AET) and serotonin have been found to cause a rapid fall in tissue NAD levels. The results have been briefly discussed.**

THE continuous counting of $^{32}P$ from tumours by way of an embedded Geiger counter was first described by Hale (1961). Later, Bullen, Freundlich, Hale, Marshall and Tudway (1963) concluded from clinical experience with the technique that a peak in $^{32}P$ activity coincided with a period of relative radiosensitivity whilst a trough in activity coincided with a period of relative radioinsensitivity. Studies with experimental tumours in animals were made by Bleehen, Bryant and Gallear (1967), Calcutt, Bullen, Marshall and Godden (1967) and Woolley-Hart, Twentyman, Corfield, Joslin, Morrison and Fowler (1968). All these groups found fluctuations to occur in $^{32}P$ counting rates from tumours. Whilst the exact nature of these fluctuations has not been identified Taylor, Parker, Field and Greatorex (1968) have concluded that they are metabolic in origin.

Extension of this work has now led to the discovery that compounds inducing a new synthesis of nicotinamide adenine dinucleotide (NAD, diphosphopyridine nucleotide or coenzyme I) will induce peaks in the $^{32}P$ counting rate from tumours. This finding has allowed experimental test of the clinical impression of radiosensitivity at the times of such peaks.

### *$^{32}P$ uptake studies*

Mice of either sex implanted with transplantable tumours have been used in conjunction with B.I.N. (Twentieth Century Electronics) miniature Geiger counters. Instrumentation was as described by Calcutt *et al.* (1967). Each animal was given $^{32}P$ by intraperitoneal injection of a solution of radioactive sodium phosphate in distilled water at the rate of 1 $\mu$Ci per 100 g. of body weight. Further experiments were only carried out when a stable trace had been obtained for at least 24 hours.

During a survey of compounds of physiologic importance it was found that nicotinic acid in high doses (200–600 mg./kg.) caused transient rises in the $^{32}P$ count some 3–4 hours after intraperitoneal injection. That this peaking in the $^{32}P$ count was due to the known vasodilatory action of nicotinic acid seemed unlikely in view of the relatively long time delay. This explanation was discarded finally when it was found that the related—but non-vasodilatory—agent nicotinamide caused a similar response in the $^{32}P$ count. Extension of this work resulted in the finding that the anti-vitamin 3 acetylpyridine also caused peaks in the $^{32}P$ traces. On the other hand the alternate anti-vitamin 6 aminonicotinamide was found to have no effects whatever on $^{32}P$ traces when tested against a series of different tumours. It was noticeable in these studies that response in the $^{32}P$ trace was not invariable. It actually occurred in some two-thirds of the tumours tested and to date the occurrence or otherwise of response is still unpredictable. The time at which any response occurred was somewhat variable. Usually about 3–4 hours after injection of the eliciting agent it could on occasion be delayed or even occur earlier.

*Tumour NAD levels*

The three agents found successful in eliciting peaks in the $^{32}P$ count rates are all known to cause a new synthesis of NAD (see review by Shuster, Langham, Kaplan and Goldin (1958)) whilst the fourth—6 amino-nicotinamide—which was inactive was found by Shapiro, Dietrich and Shils (1957) to have no effects on tissue NAD levels. The possibility of an association between tumour phosphate and tumour NAD levels after treatment with agents causing new synthesis of NAD was examined. Measurements of NAD by the enzymatic technique of

FIG. 1.—The response of Harding-Passey melanoma to 3 acetylpyridine (60 mg./kg.) by intraperitoneal injection at time indicated by arrow.
A—$^{32}P$ trace from tumour by way of embedded Geiger counter. Parallel lines indicate ±3 standard deviations of local mean count rate.
B—Tumour NAD levels.
Read from right to left.

Klingenberg (1965) in tumours from animals pretreated with nicotinamide, nicotinic acid or 3-acetylpyridine showed that maximal levels of NAD occurred at the times of $^{32}P$ peaks, or a little earlier. Some results are illustrated in Fig. 1. Similar findings have been made with skin carcinoma P.L. 64, sarcoma Bp. 64/12 and the Crocker sarcoma S.180.

*Radiosensitisation experiments*

In an initial series of experiments whole body sensitisation was investigated. Using 14-week-old BALB/c mice an $LD_{50/30}$ of 715 rads of $^{60}Co$ gamma rays was found. A further 120 mice were then treated in groups of 20 with single doses of $^{60}Co$ gamma rays above and below this $LD_{50/30}$ dose. Half of each group of animals was given a single dose of nicotinamide by intraperitoneal injection 3½ hours before irradiation. The $LD_{50/30}$ was calculated from a probit plot of the surviving fraction from each group. The lowering of the $LD_{50/30}$ by pretreatment with nicotinamide is shown in Table I, together with data showing

TABLE I.—*The Response of BALB/c Mice to Whole Body Irradiation 3½ Hours After Pretreatment with Nicotinamide*

| Dose of nicotinamide (mg./kg.) | Change in $LD_{50}$ (rads) | Ratio of weight loss $\frac{\text{treated}}{\text{controls}}$ at $LD_{50}$ after 8 days |
|---|---|---|
| 200 | −35 | 1·40 |
| 400 | −65 | 1·65 |
| 600 | −50 | 1·60 |
| 800 | −45 | 1·53 |

an increased post irradiation weight loss in the pretreated animals. Irradiated control mice lost about 2·5 g. (10% body weight) at 8 days post irradiation and about 8·0 g. (35% body weight) just before death. Whilst the reductions in $LD_{50}$ and the increased weight losses appear consistent with one another they do not appear to be consistent with the increased doses of nicotinamide. They do, however, appear consistent with the level of induced NAD since Shuster *et al.* (1958) have found NAD synthesis to increase with increasing nicotinamide dosage to a maximum with a nicotinamide dosage of 450 mg/kg. Further increase in the nicotinamide dosage leads to a decline in NAD synthesis.

That the above results were not due to a toxic action of the nicotinamide is shown by the fact that nicotinamide at 450 mg./kg. causes a slight increase in the weight of treated mice as compared with untreated controls.

Tumour sensitisation has been investigated with Crocker S.180 tumours transplanted in the flanks of BALB/c mice. Pairs of tumours matched for size (less than 250 $mm^3$) were selected and one animal was given nicotinamide (400 mg./kg.) by intraperitoneal injection. The paired tumours were irradiated 3½ hours later with 95 kV X-rays at 165 rad/min. Body shielding was with 4 mm. lead in the form of a tube and the tumour was pulled through a slot in the lead. Twenty-five pairs of tumours have been treated in this fashion with doses ranging from 3 to 5·2 krads, each dose being given in equal fractions in two or three different axes. Of the 25 control tumours four regressed completely, four persisted without growth for 21 days and the remainder renewed growth after a temporary check. Of the 25 pretreated with nicotinamide 24 regressed completely leaving a scar whilst one grew without check. This last result may have been the consequence of a laboratory error in coding the animals. Two animals from this series have since shown further tumour growth, in each case in an unirradiated area. Both cases have probably arisen from tumour fragments left in the trochar track during the original transplantation. Currently, some animals have survived more than 6 months with no recurrence of their tumour. The tumour growth patterns of a pair of tumours and of one completely untreated tumour are shown in Fig. 2. The appearance of one experiment 21 days after irradiation is illustrated in Fig. 3.

FIG. 2.—Growth rates of sarcoma S.180.
A—Untreated tumour.
B—Tumour treated with 4·5 krads.
C—Tumour treated with nicotinamide (400 mg./kg.) $3\frac{1}{2}$ hours before 4·5 krads.

A series of experiments in which nicotinamide was given immediately before irradiation showed that treatment at this time had no apparent effect on the ultimate response to irradiation.

*Sensitisation to cytotoxic drugs*

Because of the similarities in effects of irradiation and certain cytotoxic compounds the possibility of sensitisation to cytotoxic drugs has been examined. Initial experiments with the NK lymphoma (ascites) in BALB/c mice and used 5 days after transplant of $10^6$ cells showed that sensitisation occurred $3\frac{1}{2}$ hours after treatment with nicotinamide (600 mg./kg.), 3 acetylpyridine (60 mg/kg.) or nicotinic acid (400 mg./kg.). Results in the case of nitrogen mustard ($HN_2$) and nicotinamide are illustrated in Fig. 4. This also shows that nicotinamide alone causes an increase in tumour growth as compared with untreated controls, and that at the maximum tolerated dose (3 mg./kg.) there is a reversal of the effect and some apparent protection. This happening has occurred in about one third of our experiments and is, so far, unexplained.

FIG. 4.—Dose response curve of NK lymphoma treated with nitrogen mustard ($HN_2$) 5 days after transplant.

A—Normal tumour.

B—Normal tumour treated 3½ hours previously with nicotinamide (600 mg./kg.).

Experiments in which the nicotinamide was given at other times (0–5 hours) before treatment with nitrogen mustard showed very little or no alteration in the response. On the other hand nicotinamide (600 mg./kg.) given up to 3 hours after the nitrogen mustard has resulted in an apparent protection. This, however, may not be a true post treatment protection but merely enhanced growth of residual undamaged cells induced by nicotinamide treatment.

Further work has resulted in the sensitisation of the NK lymphoma (ascites), sarcoma S.180 and skin carcinoma P.L.64 against nitrogen mustard, thiotepa and 2-amino-cyclopentanecarboxylic acid (A.C.P.C.). No effects on the response to methotrexate have been found. Using a line of the NK lymphoma rendered resistant (and cross resistant to many other agents) by long continued treatment with Degranol (mannomustine hydrochloride) a return to near the sensitivity of the normal tumour line was achieved by treatment with nicotinamide (600 mg./kg.) 3½ hours before giving Degranol.

The results of another experiment with the resistant line of the NK lymphoma are shown in Fig. 5. Groups of 5 mice transplanted with $10^6$ tumour cells 5 days earlier have been treated with nitrogen mustard (1·5 mg./kg.) at intervals after intraperitoneal injection of nicotinamide (450 mg./kg.). The results as measured 8 days later are compared with the tumour response in terms of new NAD synthesis. It will be seen that in this case the nicotinamide itself caused a slight

EXPLANATION OF PLATE

FIG. 3.—Appearance of mice bearing sarcoma S.180, 21 days after treatment. Tumour in both cases of 1 $cm^3$ volume at time of treatment.

A—3·0 krads irradiation.

B—3·0 krads irradiation 3½ hours after intraperitoneal injection of nicotinamide (400 mg./kg.).

3a

3b

FIG. 5.—Response of resistant line of NK lymphoma to nitrogen mustard after nicotinamide (450 mg./kg.) pretreatment.
A—Tumour response at various times after pretreatment.
B—Tumour NAD levels after treatment with nicotinamide (450 mg./kg.).

reduction in tumour growth, some sensitisation occurred and that this was closely associated in time with the maximal NAD response.

This tumour is also interesting in that it has a very high basal level of NAD.

*Experiments with radioprotective agents*

Since Calcutt *et al.* (1967) showed that the powerful radioprotectors, cysteamine and 2-aminoethylisothiouronium bromide (AET) will cause rapid falls in $^{32}P$ counting rates from tumours at times covering the period of maximal protection the effect of these compounds on tissue NAD levels has been examined. Using the technique of Klingenberg (1965) NAD has been estimated in the livers of rats and mice at various short intervals after giving radioprotective doses of cysteamine, AET or serotonin (5-hydroxytryptamine). All three have been found to cause a rapid and profound fall in NAD levels. Cysteamine has also been found to cause a rapid loss of NAD in rat spleen. Results for cysteamine on rat and mouse liver are shown in Fig. 6 where it will be noticed that the effect is more pronounced in mouse than in rat liver, which may be associated with the fact that cysteamine is a better protector of mice than rats (see Bacq, 1965, p. 130).

### DISCUSSION

Although the results given above only represent an interim report on work still in progress they are already so well defined as to merit further consideration.

FIG. 6.—Liver NAD levels after intraperitoneal cysteamine (150 mg./kg.). A—Rat liver—control 180±16 $\mu$g. NAD per 1 g. wet weight of liver. B—Mouse liver—control 175±33 $\mu$g. NAD per 1 g. wet weight of liver.

The three agents acting as sensitisers appear to do so by indirect action of inducing synthesis of new NAD and since sensitisation has only been found at the peak of the new NAD synthesis it would seem that the NAD is the proximate sensitiser. This conclusion is supported by the finding that the potent radio-protective agents cysteamine, AET and serotonin all cause an immediate loss of NAD. The period during which NAD is low is the one during which radio-protection is maximal. To date we have been unable to find any association between normal tissue levels of NAD and the response to irradiation or drugs. It appears that a change from the level of the normal balanced system of cellular biochemistry is essential in order to influence the response to irradiation or drugs. This question is being investigated in further detail.

There are two further points which are in keeping with a direct role for NAD in the response to external agents. Land and Swallow (1968) have shown that NAD can be reduced by hydrated electrons and that a very rapid and efficient intramolecular electron transfer occurs. Since under *in vivo* conditions a considerable amount of NAD exists in association with proteins the possibility of further intermolecular transfer cannot be discounted. Thus the absolute amount of NAD could influence the extent of primary damage. Secondly, NAD is the requisite coenzyme for many oxidative systems, so a change in NAD levels would be expected to influence metabolic processes and consequently affect the extent of fixation of prior damage, or equally, to affect recovery processes.

The nature of the relationship between changes in NAD levels and the apparently associated changes in phosphate levels, as indicated by $^{32}P$ counts, remains a matter of conjecture. Certainly, the actual amounts of phosphate involved are very much greater than can be accounted for as the phosphate groups of the NAD concerned. The phosphate level changes although, as previously mentioned, unpredictable in their occurrence have proved an adequate indication of the timing of the NAD changes and have allowed the setting up of an experimental system to test for radiosensitisation at the times of peak activity of phosphorus. The success of these experiments makes it appear that the opinion of Bullen *et al.* (1963) based on clinical data and spontaneous phosphorus level changes, that a peak in $^{32}P$ counting rates from tumours represents a time of radiosensitivity could be well founded.

The role of sulphydryl (–SH) groups in determining the response to irradiation (Bacq, 1965) or to cytotoxic drugs (Calcutt and Connors, 1963) has been the subject of much inconclusive work in the past. With the current demonstration that cysteamine and AET, both known to affect cellular –SH levels during the period of protection, also affect cellular NAD at the same time the problem of interrelationships between –SH and NAD is raised. The associated changes in phosphate levels must also be considered. Although lack of evidence precludes further discussion now this situation emphasises the need to consider cell biochemistry as an integrated system rather than in terms of isolated entities.

Both nicotinic acid and nicotinamide are freely available, widely used pharmaceuticals which are tolerated in large doses by human beings. In the light of the above experimental findings it is necessary to emphasise that the uncontrolled use of these or related compounds in conjunction with radiotherapy or cytotoxic drugs could lead to unexpected results.

We are indebted to Dr. R. C. Tudway of the Radiotherapy Department, Bristol General Hospital, for his encouragement and for the provision of radiation facilities. The expenses of this work have been defrayed from a block grant to Mount Vernon Hospital from the British Empire Cancer Campaign for Research and grants to Bristol General Hospital from the United Bristol Hospitals' Research Committee and Tenovus.

### REFERENCES

BACQ, Z. M.—(1965) ' Chemical Protection against Ionising Radiation '. Springfield, U.S.A. (C. Thomas).

BLEEHEN, N. M., BRYANT, T. H. E. AND Gallear, R.—(1967) *Clin. Radiol.*, **18**, 237.

BULLEN, M. A., FREUNDLICH, H. P., HALE, B. T., MARSHALL, D. H. AND TUDWAY, R. C.—(1963) *Post-grad. med. J.*, **39**, 265.

CALCUTT, G., BULLEN, M. A., MARSHALL, D. H. AND GODDEN, T. J.—(1967) *Br. J. Cancer*, **21**, 438.

CALCUTT, G. AND CONNORS, T. A.—(1963) *Biochem. Pharmac.*, **12**, 839.

HALE, B. T.—(1961) *Lancet*, ii, 345.

KLINGENBERG, M.—(1965) in ' Methods of Enzymatic Analysis ', edited by Bergmeyer, H. U. London (Academic Press), p. 528.

LAND, E. J. AND SWALLOW, A. J.—(1968) *Biochim. biophys. Acta.*, **162**, 327.

SHAPIRO, D. M., DIETRICH, L. S. and SHILS, M. E.—(1957) *Cancer Res.*, **17**, 600.

SHUSTER, L., LANGHAM, T. A., KAPLAN, N. O. AND GOLDIN, A.—(1958) *Nature, Lond.*, **182**, 512.

Taylor, D. M., Parker, R. P., Field, E. O. and Greatorex, C. A.—(1968) *Br. J. Radiol.*, **41**, 432.
Woolley-Hart, Ann, Twentyman, P., Corfield, J., Joslin, C., Morrison, P. and Fowler, J. F.—(1968) *Br. J. Radiol.*, **41**, 440.